# Assessment of American Bullfrog (*Lithobates catesbeianus*) spreading in the Republic of Korea using rule learning of elementary cellular automata

**DOI:** 10.1038/s41598-024-62139-3

**Published:** 2024-05-21

**Authors:** Gyujin Oh, Yunju Wi, Hee-Jin Kang, Seung-ju Cheon, Ha-Cheol Sung, Yena Kim, Hong-Sung Jin

**Affiliations:** 1 Department of Mathematics and Statistics, Chonnam National University, 77 Yongbongro, Bukgu, Gwangju, 61186 Republic of Korea; 2 School of Biological of Sciences and Biotechnology, Chonnam National University, 77 Yongbongro, Bukgu, Gwangju, 61186 Republic of Korea; 3 Department of Biological Sciences, College of Natural Sciences, Chonnam National University, 77 Yongbongro, Bukgu, Gwangju, 61186 Republic of Korea; 4 Department of Mathematics, Hawaii Pacific University, 1 Aloha Tower Drive, Honolulu, HI 96813 USA

**Keywords:** Bullfrogs spreading, Clustering, ECA, CNN, Presence location, Habitat Suitability, Computational biology and bioinformatics, Ecology

## Abstract

The spread of American Bullfrog has a significant impact on the surrounding ecosystem. It is important to study the mechanisms of their spreading so that proper mitigation can be applied when needed. This study analyzes data from national surveys on bullfrog distribution. We divided the data into 25 regional clusters. To assess the spread within each cluster, we constructed temporal sequences of spatial distribution using the agglomerative clustering method. We employed Elementary Cellular Automata (ECA) to identify rules governing the changes in spatial patterns. Each cell in the ECA grid represents either the presence or absence of bullfrogs based on observations. For each cluster, we counted the number of presence location in the sequence to quantify spreading intensity. We used a Convolutional Neural Network (CNN) to learn the ECA rules and predict future spreading intensity by estimating the expected number of presence locations over 400 simulated generations. We incorporated environmental factors by obtaining habitat suitability maps using Maxent. We multiplied spreading intensity by habitat suitability to create an overall assessment of bullfrog invasion risk. We estimated the relative spreading assessment and classified it into four categories: rapidly spreading, slowly spreading, stable populations, and declining populations.

## Introduction

The American Bullfrog, *Lithobates catesbeianus*, has been introduced to more than 40 countries worldwide and is listed on the “100 of the World’s Worst Invasive Alien Species” ^1^. American Bullfrog was introduced to Korea in 1957 and cultivated for the purpose of establishing new food sources for human consumption, but due to its low economic efficiency and low demand as food, most farms gave up on farming and released them into rivers illegally, and bullfrogs were spread throughout the country the country^2–4^. Korea is originally an agricultural society, and even now, no crops other than rice can be grown on farmland^5,6^. Rice farming requires a lot of water, so the area around the farmland has a good environment for bullfrogs to live in, such as reservoirs, waterways, rice paddies, etc. Here, bullfrogs abandoned by farms find the best habitat and their population has rapidly increased. The negative effects of the bullfrog invasion on native species arise from competition, amphibian and fish predation, as well as the spread of ranavirus and the fungus Batrachochytrium dendrobatidis, which is systematically killing amphibians^7^. A variety of control methods are needed to prevent further invasions based on local ecology and land use^8–14^. In Korea at least 84% of native anurans (frogs and toads) were at moderate to extreme risks, which included all frogs but only 33% of toads. to set conservation priorities and strategies^15^. It is important to assess the extent of the spread of invasive species by integrating biotic and abiotic data collected at different spatial scales to assess where invasive species monitoring and management efforts should be focused focused^16^. Bullfrogs have continued to spread in an environment without natural enemies and have now spread nationwide except in some mountain areas in South Korea^14,17^. The species was reported to occur at 2716 sites, mainly distributed along the southern and western coasts, but rarely occurred in the northern part of Korea or along the eastern coast^17^. Future predictions suggest continued bullfrog spread^14^.

The Ministry of Environment and non-governmental organizations tried several approaches to eradicate L. catesbeianus populations which resulted in significant populations declines^12,18–24^. However, these actions were discontinued and populations were allowed to expand in some local regions^12^.

In this study, the intensity of spread by region was calculated using only spatial data, not temporal data. The analysis of the intensity of the spread of bullfrogs in this paper is based on decades of observational data and can be said to reflect the characteristics of each region. The biological and environmental conditions of the habitat vary from region to region and continue to change due to various socio-environmental factors. The population may temporarily decrease over time, but the population may change at any time depending on the characteristics of the region. In particular, although there is currently no significant population increase due to effective control, the population may increase rapidly in areas with high spread intensity at any time if vigilance is relaxed. Knowing the intensity of the regional spread of invasive alien species that cause changes in biodiversity is expected to be of great help in establishing and implementing management policies accordingly. Although observation data may have errors depending on the methods, the data includes environmental characteristics of the area where bullfrogs were observed and reflect many biological and ecological factors. However, it is impossible to observe a large area over time. In this paper, we estimate the intensity of regional spread only with accumulated spatial distribution data. In the process of estimating the spreading intensity, machine learning methods such as the clustering method, ECA method, and CNN method are used. Assessment of spreading is obtained by multiplying spread intensity by habitat suitability. Species distribution modeling software Maxent 3.4.1 was used to estimate habitat suitability by reflecting local environmental and ecological information^16,25^. The spreading assessments are scored by calculating the intensity of spread and habitat suitability in 25 regions. These are then classified into areas where the population is expected to continue to increase, areas where there is no significant change in the current population, and finally areas where the population is expected to decrease.

## Material and methods

Biogeographic distribution patterns of amphibians are analyzed based on the clustering method^26,27^. Since we do not have time series data of bullfrog distribution, we analyze the spatial distribution using the hierarchical divisive clustering method using scikit-learn 1.3.0^26–29^.

The entire data is clustered into small clusters, and the degree of spreading is estimated by the evolution rules from the elementary cellular automata scheme^30–32^ in each small cluster. Elementary cellular automata consist of cells with a value of 1 or 0 and are very useful for biological modeling consisting of presence or not data. It has been used for biological and ecological modeling since the 1980’s ^28^.

CNN is trained to learn the evolution rules ^33–36^. By recognizing small clusters as a single image of 0's and 1's, we count the number of 1's, representing the presence location. This allows us to define the spreading intensity as the ratio of the expected number of presence locations over 400 generations to the initial number of presence locations.

When calculating the intensity of spread using machine learning on accumulated observation data, biological and environmental factors were not reflected. We incorporated environmental factors by obtaining habitat suitability. The habitat suitability is achieved using Maxent software ^9,16,25,37–42^. Habitat suitability models can help to understand and predict the dynamics of invasions. MAXENT is a machine learning method that estimates the distribution of a species by finding the probability distribution of maximum entropy, subject to constraints representing our incomplete information about the distribution^9^. The model evaluates the suitability of each grid cell as a function of environmental variables. The estimated spreading intensity is multiplied by the habitat suitability to express the assessment of bullfrog spreading by region.

### Observation data

**Figure 1:**
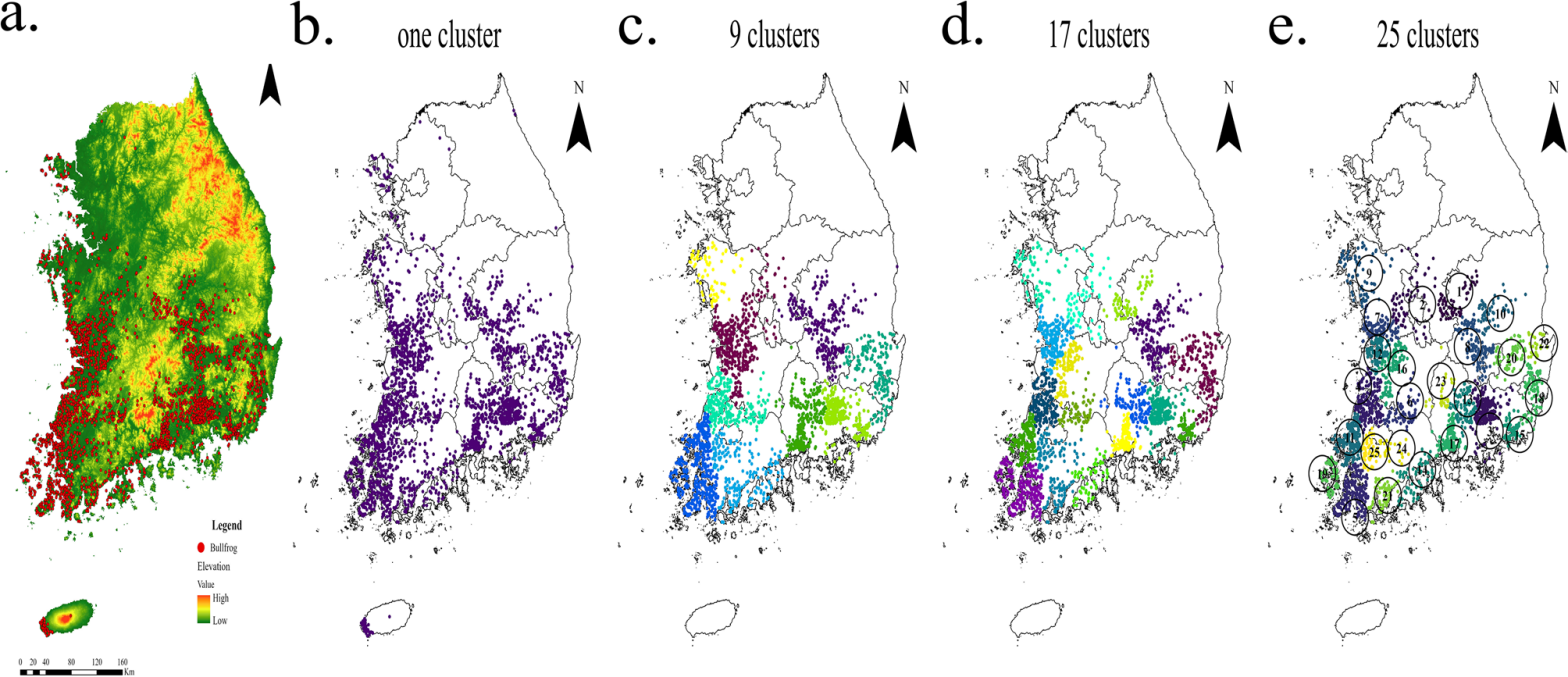
Distribution of bullfrog observations and results of divisive clustering. (**a**) The map above represents South Korea, and the data is between latitude 34° 58ʹ–36° 71ʹ and longitude 126° 11ʹ–128° 2ʹ, covering approximately the southern half of South Korea. It shows where bullfrogs have been found on the topographic map. The highest elevations are red, then moving to orange, yellow, bright greens, and finally dull greens at the lower elevations. It is mainly distributed in coastal wetlands or riverside wetlands and is rarely distributed in mountainous areas. This is a collection of findings over 60 years, with lacking temporal information (**b**) Observation data (**c**) divisive clustering after 9 clusters are formed (**d**) divisive clustering after 17 clusters are formed (**e**) the size of the clusters became similar to the size of the administrative district at 25 clusters. The maps were generated using ArcGIS Pro 3.1.1 (ESRI, USA), esrikr.com.

All data is collected from the results of 3^rd^ investigation of natural environment from 2006 to 2012, the National Wetland Center Report from 2011 to 2017, and the National Institute of Ecology from 2015 to 2017^17^ (See the Supporting Documents and Tables S1, Tables S2). The surveys were conducted by amphibian and reptile experts between January and December of each year. For amphibian identification, daytime observations using fish pots, skimming nets, and visual observations of adults were conducted. Acoustic surveys for amphibian calls were conducted at night. Figure 1a shows the distribution of American Bullfrogs observed in recent decades in South Korea. Bullfrogs have rarely been observed in mountainous and urban areas. There is no time series data for the study area.

The Republic of Korea is a type of mountainous country rarely seen throughout the world and its mountainous area covers more than 70% of the land. Mountains in general are high to the north and to the east, and low to the west and to the south with the ridge of the spine lying inclined toward the east to form steep slopes along the east coast and slow slopes along the west coast in Fig. 1a ^43^.

### Clustering

To estimate the intensity of spreading by region, a sequence of spatial distribution from the observed data in Fig. 1a is constructed using the divisive hierarchical clustering method. All observations start in one cluster of full data, and splits are performed recursively as one moves down the hierarchy by grouping neighboring data into the same cluster^27^. The scikit-learn clustering software^29^ is used, and clusters are numbered according to the order in which they are formed. Clustering is performed until 25 clusters are formed to roughly match the size of the administrative district. Rectangular images consisting of 20 by 20 cells are created by uniformly dividing the latitude and longitude including all observations in each cluster. If each cell had a bullfrog observation point, it is marked as 1, otherwise it is marked as 0. Here, the point density of each cell is inhomogeneous. Some cells have one observation point and some have many points. Those cells are equally treated as presence points. We assumed that bullfrogs had never been found outside of the cluster. In each rectangular image, some cells are not included in the cluster, and the corresponding cell value is assumed to be 0. Latitude and longitude information for all clusters is in Table 1. In Maxent Software, it is handled differently when using presence/absence data and when using presence-only data. It is recommended the logistic option for presence-only data, and the cloglog option for presence/absence data^25^. Hence, the cloglog (default in Maxent 3.4.1) option is used to treat occurrence records as points rather than grid cells to estimate relative habitat suitability^25^.

**Table 1:** The results of bullfrog spreading for 25 clusters.

Clustering number	Number of presence location	SI	HS	SA	Longitude	Latitude
1	77	3.25	0.331892778	1.078651528	127.99	36.42
2	83	2.87	0.336620682	0.966101357	127.27	36.42
3	267	3.1	0.772864513	2.395879991	128.47	35.36
4	202	1	0.740367631	0.740367631	126.64	35.47
5	168	2.48	0.794083093	1.96932607	126.43	34.58
6	103	1.51	0.594828446	0.898190953	127.2	35.36
7	127	3.25	0.713751758	2.319693214	126.77	36.16
8	125	3.24	0.65006152	2.106199325	128.33	36
9	96	2.08	0.460205108	0.957226626	126.47	36.71
10	58	3.46	0.473968133	1.639929741	128.6	36.33
11	205	1.26	0.791182732	0.996890243	126.46	35.08
12	135	3.25	0.781342992	2.539364724	126.76	35.93
13	98	3.42	0.62174352	2.12636284	128.14	35.4
14	68	2.23	0.634174926	1.414210085	127.36	34.73
15	87	1.35	0.690117529	0.931658665	128.88	35.18
16	113	3.16	0.673967381	2.129736922	127.01	35.78
17	136	2.91	0.701436489	2.041180182	127.91	35.11
18	107	1.1	0.62500829	0.687509118	129.21	35.57
19	70	2.66	0.697356021	1.854967015	126.11	34.73
20	65	2.86	0.6193504	1.771342144	128.88	35.89
21	58	2.14	0.617724528	1.321930491	126.88	34.58
22	30	3.33	0.573416667	1.9094775	129.28	35.99
23	37	2.02	0.3649146	0.737127492	127.81	35.53
24	37	2.21	0.575529162	1.271919448	127.03	34.99
25	65	2.22	0.804474672	1.785933772	126.66	34.98

The final spreading assessment is the spreading intensity multiplied by the habitat suitability estimated by Maxent software 3.4.1. The higher the value, the greater the probability of spreading.

**Figure 2:**
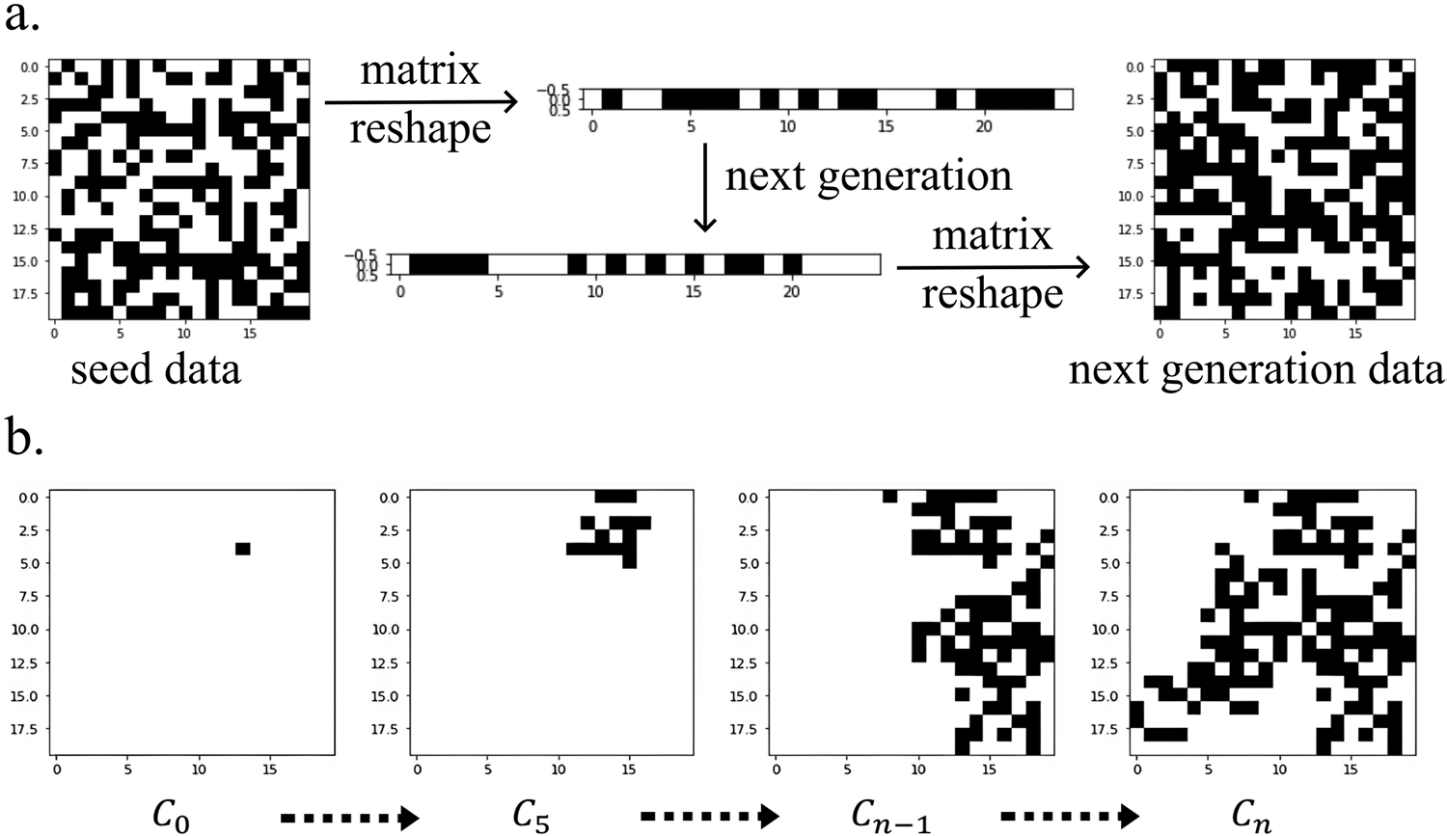
Training the ECA rules and generate image sequences for each cluster. (**a**) Reshape the 20 by 20 matrix to a 1 by 400 matrix, then apply the elementary cellular automata rules to generate the new generation of a 1 by 400 matrix. Reshape the new matrix to 2 dimensional 20 by 20 matrix. To train the ECA rules, we generate 1500 matrix pairs for all possible even rules of ECA by random seeding of 1’s at 100, 200, and 300 initial points we generate. (**b**) The image sequence, ${{\text{C}}}_{0}\to {\cdots {\text{C}}}_{5}\to \cdots {{\text{C}}}_{{\text{n}}-1}\to {{\text{C}}}_{{\text{n}}}$, is created by applying the agglomerate clustering method. Estimate the distribution of rule from ${{\text{C}}}_{{\text{n}}-1}$ to ${{\text{C}}}_{{\text{n}}}$.

To estimate the spreading intensity of each cluster the agglomerate clustering method is performed in each cluster making the sequence of images, ${C}_{0}\to {C}_{1}\to \cdots {C}_{n-1}\to {C}_{n}$. Figure 2b illustrates the agglomerate clustering steps, taking cluster #5 in Fig. 1e as an example.

### Learning elementary cellular automata rules

ECA is introduced to find rules in the sequences for each cluster. ECA is a one-dimensional array of cells, where each cell takes either 1 or 0, representing presence or not presence, respectively. It generates the next array depending on its state and the states of its two closest neighbors^30,31,44,45^. Hence, 256 rules numbering from 0 to 255 are available to represent the sequence evolution. In this study, only the even number rules are used. The odd number rules are excluded because it makes the next generation value 1 when both the current cell and the neighboring cells are 0, so the bullfrog appears after not being present, which is not suitable for the biological spreading model. In extreme cases, when a bullfrog is found in only one location, applying the odd number rule results in bullfrogs being found in the entire cell in the next generation. Each cell in an ECA row represents one generation, where ECA is a one-dimensional array. The next generation is generated by the ECA rules. By reconstructing a one-dimensional array into a 2D image, each generation can be made of a sequence of images that change according to the ECA rules in Fig. 2a.

The rules are learned by training the image change pattern using the Convolutional Neural Network(CNN) ^46,47^. CNNs are a subset of a class of deep learning algorithms, most commonly used for spatial pattern analysis in biology and ecology^33,36,47^. Additionally, CNN methods can efficiently classify the predicted distributions of many species^35^. In this simulation CNNs are trained with the Keras package in TensorFlow^48^.

#### Generate training data

The procedure is as follows:

- Create a 20 by 20 matrix by random seeding of 1’s at 100, 200, and 300 initial points.
- Reshape the 20 by 20 matrix to a 1 by 400 matrix
- Generate the next generation of 1 by 400 matrix according to ECA rules.
- Reshape two consecutive 1D matrices to two consecutive 2D matrices in Fig. 2a, which are considered as one set of images, such as $({C}_{n-1}, {C}_{n})$ in Fig. 2b.
- Generate sets of image data for all 128 possible even rules
- Generate 500 sets of image data for each 100, 200, and 300 initial points for each rule

Hence, 500*3*128 = 192,000 sets of image data are generated.

#### Training the rules

- Separate 80% of training data and 20% of test data from total data
- Learning the rules using CNN(Convolution Neural Network) method

### Spreading intensity

To estimate the intensity of spreading, the expected number of presence location variations depending on the rules governing the evolution of clusters is estimated. As an initial value, a value of 1 is randomly given to 100 cells out of 400 cells of the image, and then the number of 1 s in the image is counted while evolving over 400 generations according to all even-number rules of ECA. This process is repeated 10 times to get the expected number of 1 s. The expected number of presence locations over 400 generations with the initial value set to 100 for the rule 204, 206 and 220 is shown in Fig. 2b–d respectively. The mean of the expected number of presence location over 400 generations divided by the initial value of 100 is defined as the spreading intensity for each rule, which shows the growth rate of the expected number of presence locations. The results for all possible even rules are in Fig. S2. The mean of the expected number of presence locations according to each rule is multiplied by the percentile distribution of the rules to get the mean of the expected number of presence locations of the cluster. Here the *spreading intensity* is defined as the mean of the number of presence locations:

$$spreading intensity =\sum_{i}\mathrm{Percentile of rule }\left({{\text{x}}}_{i} \right)*\mathrm{ mean of the expected number of presence location for rule }({{\text{x}}}_{i} )/100$$

### Assessment of spreading

Since the spreading intensity is evaluated based on the mean of the expected number of presence locations only without considering any other environmental and biological variables, the final predicted spreading intensity is weighted by the habitat suitability. The Maxent software (Maximum Entropy, version 3.4.1) is used in estimating the relative habitat suitability of sites by comparing environmental conditions at known observed sites to the available environmental conditions such as precipitation, temperature, elevation, and so on^40,41^. In this paper, correlation analysis between variables was applied using multicollinearity. As a result, 6 main environmental factors out of 19 factors were used, but there may be cases where the remaining factors should not be overlooked. A pairwise Pearson correlation was performed and highly correlated variables (|r| > 0.80) were excluded, to avoid collinearity in statistical models^49^. The main environmental factors when using Maxent software are annual mean temperature, mean diurnal range, temperature seasonality, annual precipitation, precipitation of wettest month, and precipitation of driest month.

## Simulation results

### Clustering

Using the hierarchical clustering method, the entire data is divided into 25 small clusters, and the size of the clusters became similar to the size of the administrative district in Fig. 1e. The number of clusters can be set to 1, 9 or 17, depending on the size of the region of interest in Fig. 1b–d. The common feature of the clusters is the high density mainly around the waterside and wetland. However, the shape of the cluster alone does not represent the spreading intensity for each cluster. Biological and environmental information are not taken into account when grouping the clusters.

### Learning the rules using CNN

When the CNN was trained to learn ECA rules, the accuracy was over 99%. This would mean that the rules of change in the bullfrog distribution could be learned with very high confidence.

### Spreading intensity

**Figure 3:**
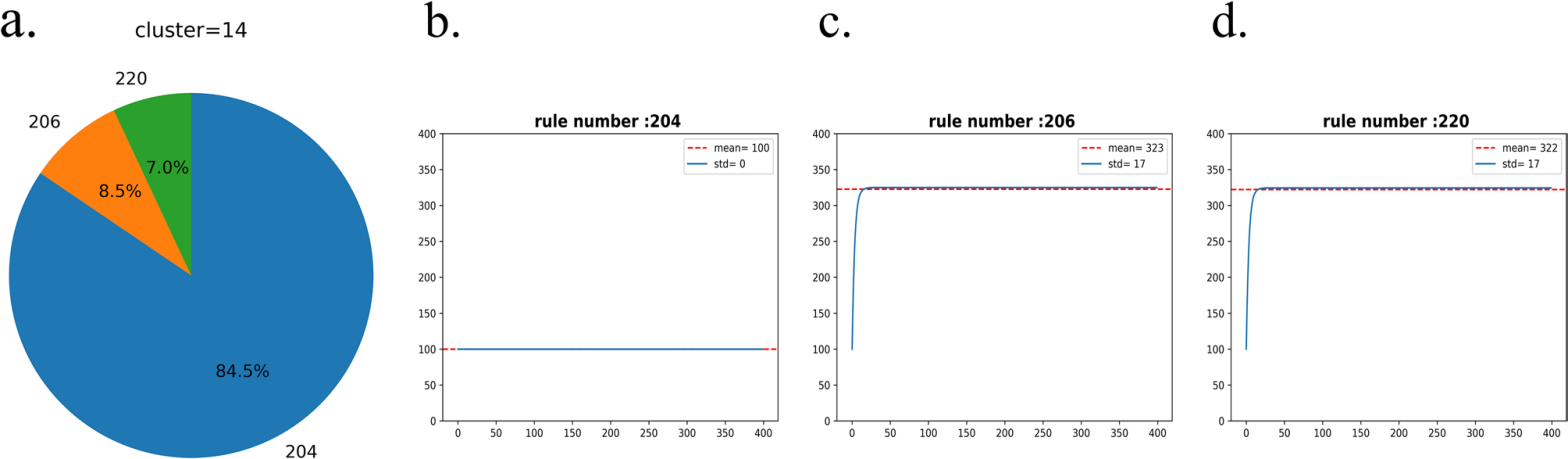
Patterns of the expected number of presence locations change by generation according to each rule. A value of 1 is randomly assigned to 100 cells out of 400 cells, and the number of cells having a value of 1 is counted up to 400 generations according to the ECA rule. (**a**) It consists of rules 204, 206, and 220 corresponding to 84.5%, 8.5%, and 7.0% respectively. (**b**–**d**) The mean of the convergent number of presence locations for rule 204 is 100, for rule 206 is 323, and for rule 220 is 322. The mean of the expected number of presence location is indicated in the legend and shown in red dotted line.

Figure S1 shows the distribution of rules predicted through CNN learning for each cluster in Fig. 1e. The expected number of presence locations for all 128 ECA rules estimated over 400 generations are in Fig. S2. Cluster 14 as an example, shows that 84.5% of the clusters are predicted to follow rule 204, 8.5% are predicted to follow rule 206, and 7.0% are predicted to follow rule 220 in Fig. 3b–d. The mean of the convergent number for rule 204 is 100, for the rule 206 is 323, and for the rule 220 is 322. Therefore, if bullfrogs are found in 100 cells now, the expected number of converged presence locations in cluster 14 can be calculated as 1.00*0.845 + 3.23*0.085 + 3.22*0.07 = 1.34495, which is the spreading intensity for the cluster 14. The spreading intensity of all clusters is shown in Table 1.

### Spreading assessment

**Figure 4:**
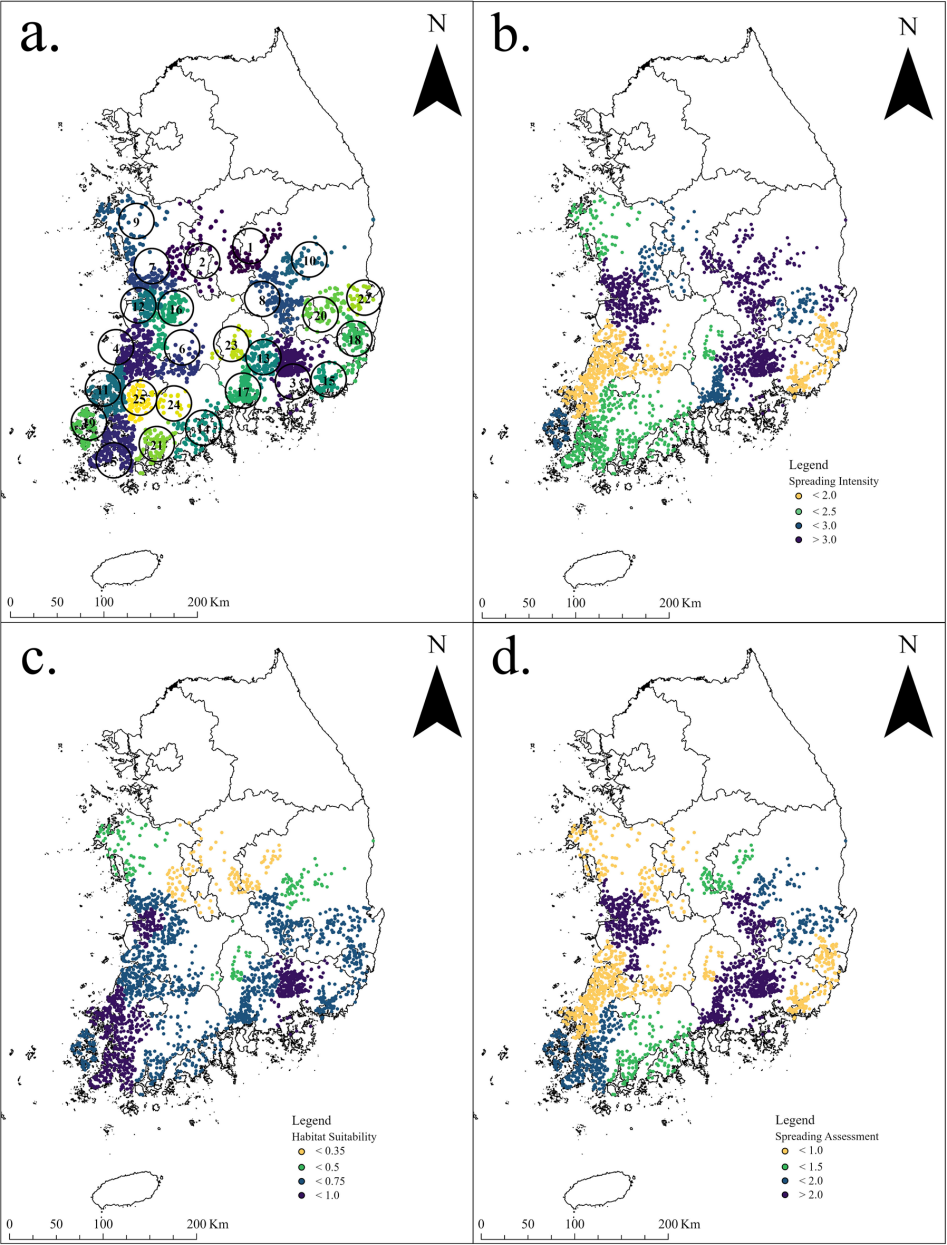
Results of 25 clusters. (**a**) 25 Clusters : Divisive clustering is performed until the clustering becames similar to the local administrative districts. (**b**) Spreading Intensity(SI): It does not show a strong spreading intensity in coastal and wetland areas. This suggests the possibility that spreading has already occurred to saturation. (**c**) Habitat Suitability(HS): Habitat suitability is calculated using Maxent software. If the SI, the spreading intensity, is weak at a high HS, it means that spreading has already occurred sufficiently. (**d**) Spreading Assessment (SA): Areas with a high probability of spreading are marked with red dots. The maps were generated using ArcGIS Pro 3.1.1 (ESRI; USA), esrikr.com.

Figure 4 shows the Spreading Intensity (SI), Habitat Suitability (HS), and Spreading Assessment (SA) of 25 clusters. Figure 4b shows the SI distribution. It does not reflect environmental and biological variables, and it shows the spreading intensity calculated only by machine learning methods (clustering, CNN, etc.). Areas that are already saturated may have low SI values, and areas with low saturation, such as mountainous areas, may have large SI values. Figure 4c represents HS distributions. Habitat suitability values obtained using Maxent software reflect ecological environmental factors for the bullfrogs. Figure 4d shows the distribution of SA values ​​obtained by multiplying SI values and HS values. The HS value ranges from 0 to 1, and the closer it is to 1, the more suitable. The spreading assessment values are categorized into four relative stages: strong spreading, weak spreading, strong retention, and weak retention.

Table 1 shows the result of calculating the spreading assessment. From left to right, each column represents the number of presence locations per cluster, spreading intensity, habitat suitability, spreading assessment, and geometric center latitude and longitude. The higher the value, the greater the probability of spreading. SI is a value calculated using machine learning based only on presence location data. Environmental and biological factors were reflected through Habitat Suitability (HS) to get a Spreading Assessment (SA).

Table 2 shows relative spreading assessments. Four cluster groups are created based on assessment scores. The clusters in groups (I) and (II) show spreading assessment scores greater than 2, which means that they will continue to spread. Clusters in group (III) show scores of 1 to 1.5, which can be considered as slow-spreading or maintaining the population. For group (IV) clusters, the spread appears to have stopped, and the population may decline, especially in clusters #4, #8, and #23.

**Table 2:** Groups of spreading assessment.

Group	Spreading assessment (SA)	Cluster number(#)	Relative results
Group (I)	2.0 < SA	3, 7, 8, 12, 13, 16, 17	Continue to spread intensively
Group (II)	1.5 < SA < 2.0	5, 10, 19, 20, 22, 25	Continue to spread
Group (III)	1 < SA < 1.5	1, 14, 21, 24	Maintain population
Group (IV)	SA < 1	2, 4, 6, 9, 11, 15, 18, 23	Maintain population and possibly decrease in 4, 18, 23

## Discussion

The study relies on static data from several surveys spanning different year but lacks true time series information to accurately track and model bullfrog spread. Obtaining time series data on bullfrogs requires a lot of manpower and budget, and in particular, it takes decades to obtain national distribution data, and it is almost impossible to obtain time series data especially when the distribution changes every year. If a space series is obtained using the unsupervised learning clustering method proposed in this paper, the intensity of spread by region can be estimated, but other observation methods must be continuously used to justify the results or prove the accuracy of the prediction. One way would be to select an area with strong spreading intensity and create time series data through continuous observation.

Methods in this study can be compared and modified using different data sources and various species distribution modeling methods. There are many sources of the occurrence data of L. catesbeianus from different archives such as GBIF, HerpNet^49^. Many spatial distribution models have been used in various combinations incorporating environmental and biological factors more comprehensively^41,49–51^. To evaluate projected range changes of L. catesbeianus in potentially suitable areas under current and future climate conditions, Johovic et al.^49^ used several algorithms combined. When implementing clustering, cases where areas with different biological and environmental conditions could not be grouped into the same cluster were not taken into consideration. Using constrained clustering, which uses biological and environmental factors as a limiting condition, will produce more meaningful results^52^. The number of clusters can be determined by determining the size of the area to be studied according to topographical, environmental, ecological, and biological characteristics.

If the number of clusters is changed, the spreading intensity, habitat suitability and spreading assessment is also changed, so the number of clusters should be adjusted to properly include the region of interest. Considering that local governments are responsible for habitat management, the size and number of clusters were determined according to the size of the administrative district.

When learning the rule, 20 by 20 cells with a maximum of 300 1 s were used, so if the number of clusters was selected when the maximum number of presence locations was less than 300, approximately 25 was appropriate. For 25 clusters the maximum number of presence locations is 267 in Table 1. Furthermore, if the maximum value exceeds 400, it becomes impossible to observe changes in the 20 by 20 cell image. Increasing the size of the image cells may allow for application to larger clusters. On the other hand, increasing the number of clusters may allow for observing more detailed changes in specific regions.

After dividing the observation data into 25 clusters, all presence sites are broadly categorized into four relative stages according to assessment sores, so the number of clusters can be set to around 20–30. When the number of clusters exceeds 20, the number of zero paddings is not very large because the sets in the cluster are relatively close together. Nonetheless, it can be a problem.

The size of cells is identical in each cluster. The density of observations in a cell is uniformly 0 or 1, where 1 represents the presence location. However, in each cell, the number of bullfrog observations is different. It is difficult to accurately measure the population density within a cluster. To increase precision, periodic observation is necessary and a method of observing the same point for a long time is also needed. The data used in this paper were assumed to exist if they were ever observed in a unit cell.

In this study, the numpy. reshape() function^53^ was used to rearrange two-dimensional images into a one-dimensional array. Future studies are needed to explore applying ECA with various array arrangements. When applying the ECA rules, zero padding was applied to both endpoints, that is, zeros are used for the cells at positions − 1 and 401 virtual cells. It is assumed that Bullfrog has been never found outside the cluster. If found, they should be included in other clusters.

In estimating spreading intensity, the mean of the expected number of presence locations is used, but the slope can be more useful in expressing the tendency of spreading in Fig. S2. Further research is needed to define the appropriate diffusion strength according to the variation pattern of the expected number of presence locations.

Since the spreading intensity is estimated based only on the data currently found it is relatively low in the region where spreading is already completed. Low spreading intensity may mean that it is already saturated, which is different from extinction. Alternatively, the carrying capacity may decrease from a population dynamics perspective due to the emergence of natural enemies or human quarantine.

Geographical characteristics and ecological characteristics are replaced by habitat suitability using Maxent but more detailed cultural characteristics should be applied. In addition to observations, appropriate detection methods for bullfrogs, such as eDNA method or audio recording devices, are required^13^.

In this paper, habitat suitability was used to reflect biological and environmental factors. Because the weight of habitat suitability was given directly as the value obtained by SDM, environmental and biological factors may not have been sufficiently reflected. Determining the weight of biological and environmental factors can be an important point. For example, if the habitat suitability obtained from Maxent is 0.6 or 0.8, and the spread intensity is 2 in some regions, then the spread assessment scores are 1.2 and 1.8, respectively and they are classified into the same group. There is no significant difference if the weight is directly assigned as a value. For a more accurate spreading assessment, the weights must be adjusted using an appropriate threshold function such as the sigmoid functions.

To classify the intensity of spread, we used 128 rules. It is possible to increase the number of rules, but it is still not possible to express patterns according to the overall environment variables. If environmental and biological variables are included, a clustering method with constraints must be applied from the cluster stage. In any case, continuous correction and supplementation work must be done through periodic observation, observation of specific areas, and various observation methods in parallel. Only the accuracy of machine learning is presented as a verification method. To verify its validity, it is necessary to select 3 or 4 regions and monitor the spreading intensity continuously for several years to generate time series data and compare it with the expected values from simulations.

Future work includes:

- Design the threshold function: For a more accurate spreading assessment, the weights estimated by Maxent must be adjusted using an appropriate threshold function such as the sigmoid functions.
- Comparative evaluation study on changes in SDM due to climate change and corresponding changes in regional spreading intensity
- Study on clustering techniques considering environmental, biological, and ecological factors
- Comparison study using other data and other SDM methods

## Conclusion

In this paper, we used machine learning methods to assess the spreading of bullfrogs in areas where they have been frequently observed in South Korea in recent decades. The extent to which bullfrogs continue to spread at observation sites is quantified and assessed. Since there is no time series data, the accumulated data were used to evaluate the spread of bullfrogs by creating a spatial series using machine learning. In this process, biological and environmental factors were not considered at all.

Cells where bullfrogs are found, and presence, are assigned a value of 1, and the number of 1 s in 400 cells composed of 1 s and 0 s is counted and used as the spreading index of bullfrogs. The mean of the number of presence locations over 400 generations, divided by the initial value of 100, is assumed to be a measure of spreading intensity for each rule. The spreading intensity is weighted by the percentile of the rules estimated by the CNN method. Under the above assumption, the intensity of spread by region was calculated using only cumulative occurrence data, and then the spread of American Bullfrog was assessed using habitat suitability as a weight reflecting environmental, biological, and ecological characteristics. Habitat suitability obtained from Maxent software includes environmental and biological factors, which were applied in the form of weights to the final spreading assessment. For a more accurate spreading assessment, the weights obtained from Maxent need to be adjusted using an appropriate threshold function such as the sigmoid functions. The weights should be determined taking into account the impact of habitat suitability on spreading assessment.

The spreading intensity by region was calculated using only cumulative data, and the spreading assessment was scored by weighting the spreading intensity with the habitat suitability for each region obtained from Maxent. The spreading assessment is determined by multiplying spread intensity by habitat suitability, which can be used as an indicator of the risk of bullfrog spread in each area.

This paper is not to analyze and predict distribution changes due to various factors such as climate change, but to find out changes in the assessment of the spread of bullfrogs in the area where they are currently distributed.

### Supplementary Information

Supplementary Tables.

Supplementary Figures.

## Data Availability

All data generated or analyzed during this study are included in this published article.
