# Supplementary material for: Assessment of American Bullfrog (Lithobates catesbeianus) spreading in the Republic of Korea using rule learning of elementary cellular automata
Source: Sci Rep. 2024 May 21;14:11548. doi: 10.1038/s41598-024-62139-3 (PMC11109106; doi:10.1038/s41598-024-62139-3)
Supplement: Supplementary file 2 — Supplementary Figures. [file 41598_2024_62139_MOESM2_ESM.docx]

**Supplementary Material**

**Assessment of American Bullfrog (Lithobates catesbeianus) spreading in the Republic of Korea using rule learning of elementary cellular automata**

**Gyujin Oh^1^, Yunju Wi^1^, Hee-Jin Kang^2^, Seung-ju Cheon^2^, Ha-Cheol Sung^3^, Yena Kim^4^,** **and Hong-Sung Jin^1*^**

1 Department of Mathematics & Statistics, Chonnam National University, 77 Yongbongro, Bukgu, Gwangju 61186, Republic of Korea

2 School of Biological of Sciences and Biotechnology, Chonnam National University, 77 Yongbongro, Bukgu, Gwangju 61186, Republic of Korea

3 Department of Biological Sciences, College of Natural Sciences, Chonnam National University, 77 Yongbongro, Bukgu, Gwangju 61186, Republic of Korea

4 Department of Mathematics, Hawaii Pacific University, 1 Aloha Tower Drive, Honolulu, HI 96813

***Corresponding author:** **Hong-Sung Jin** (E-mail: hjin@jnu.ac.kr)

**Supplementary Figures**


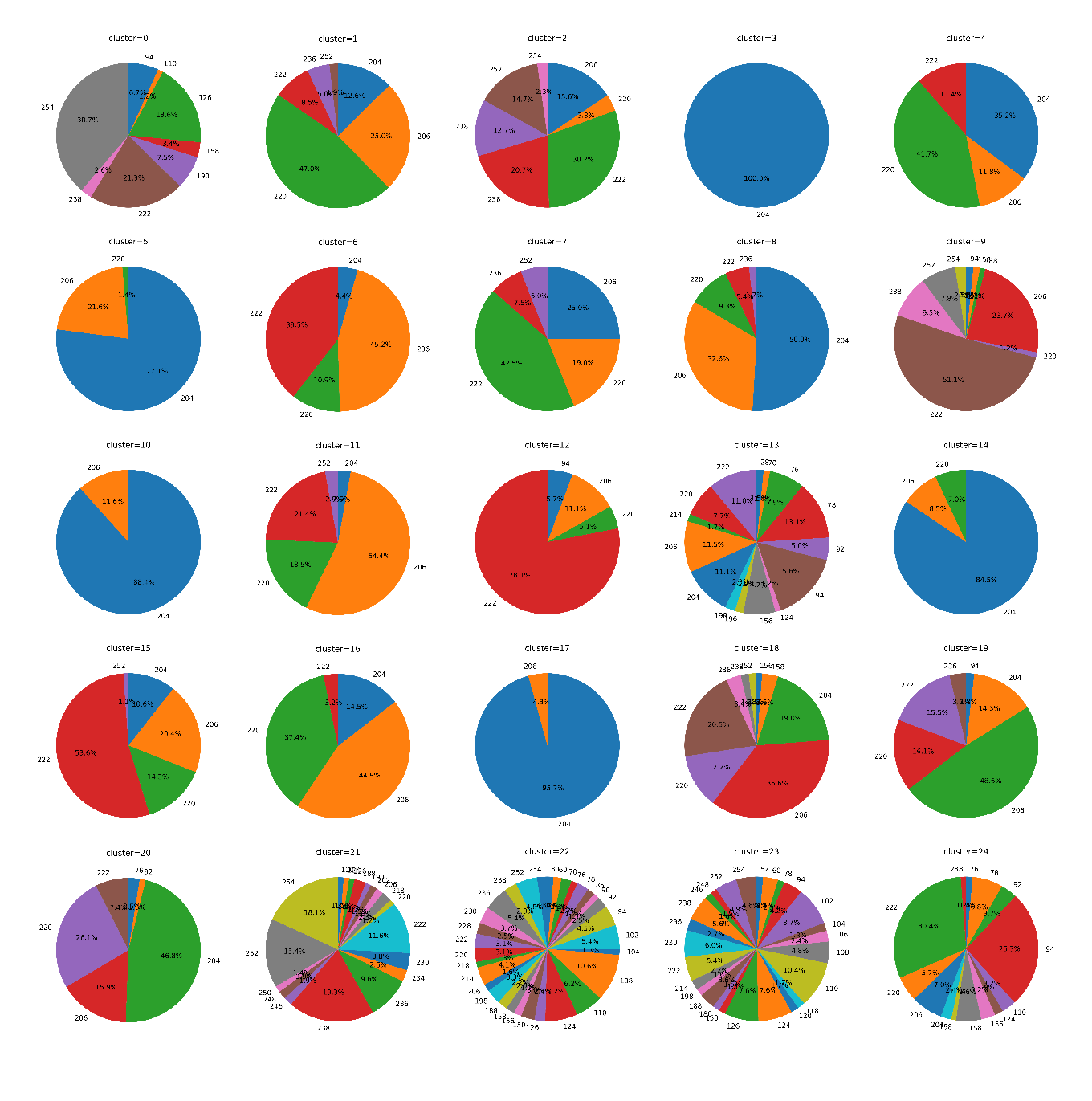


**Fig. S1** **Percentile Distribution of probabilities of predicted rules for 25 clusters.** *It shows the percentile distribution of the rules for 25 clusters. It is obtained by learning ECA rules for each cluster. The percentile distribution of the rules is obtained using the trained parameters of CNN. The CNN provides a percentile distribution for 128 rules, of which only those that occur more than 1% are shown in the circle diagram.*


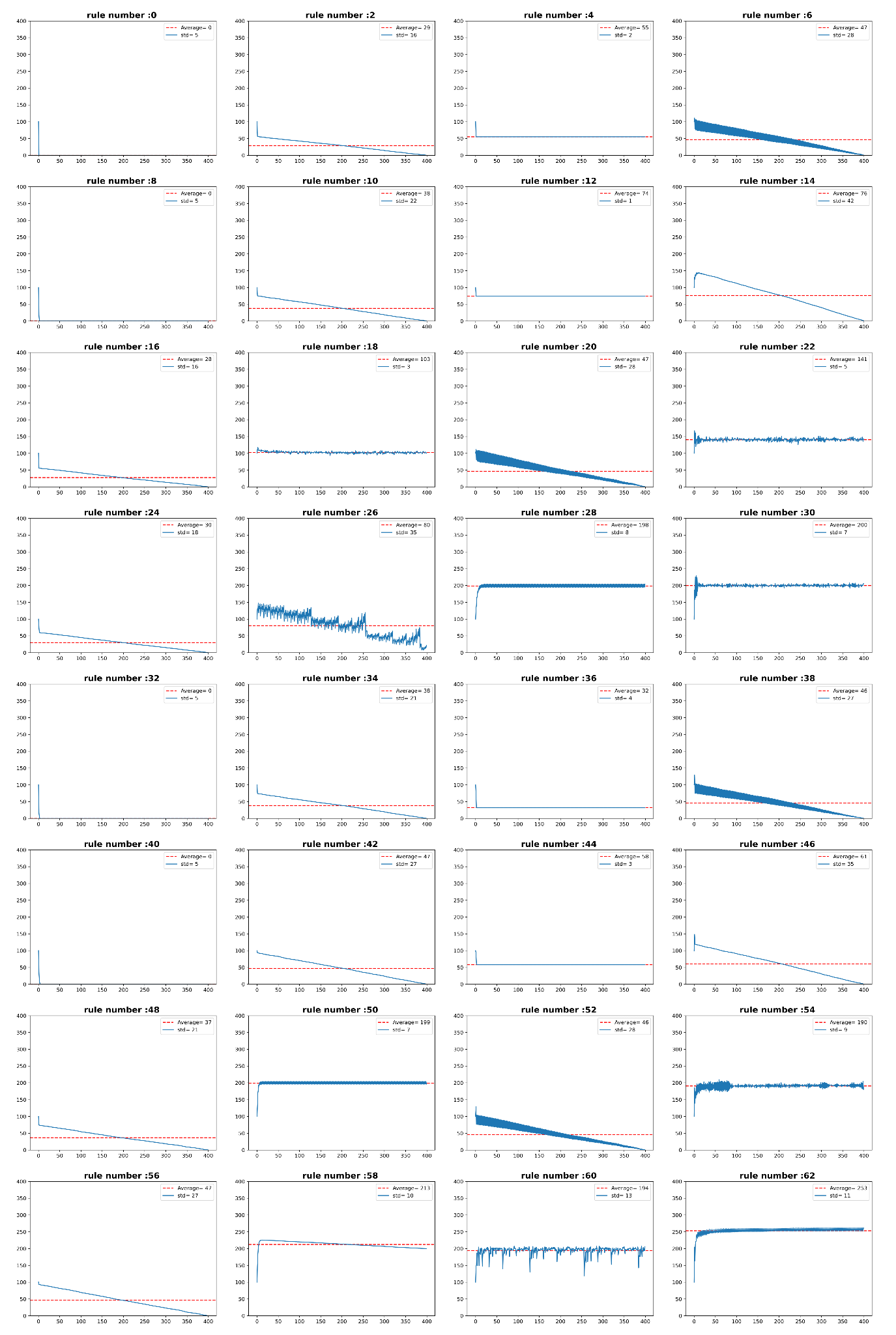


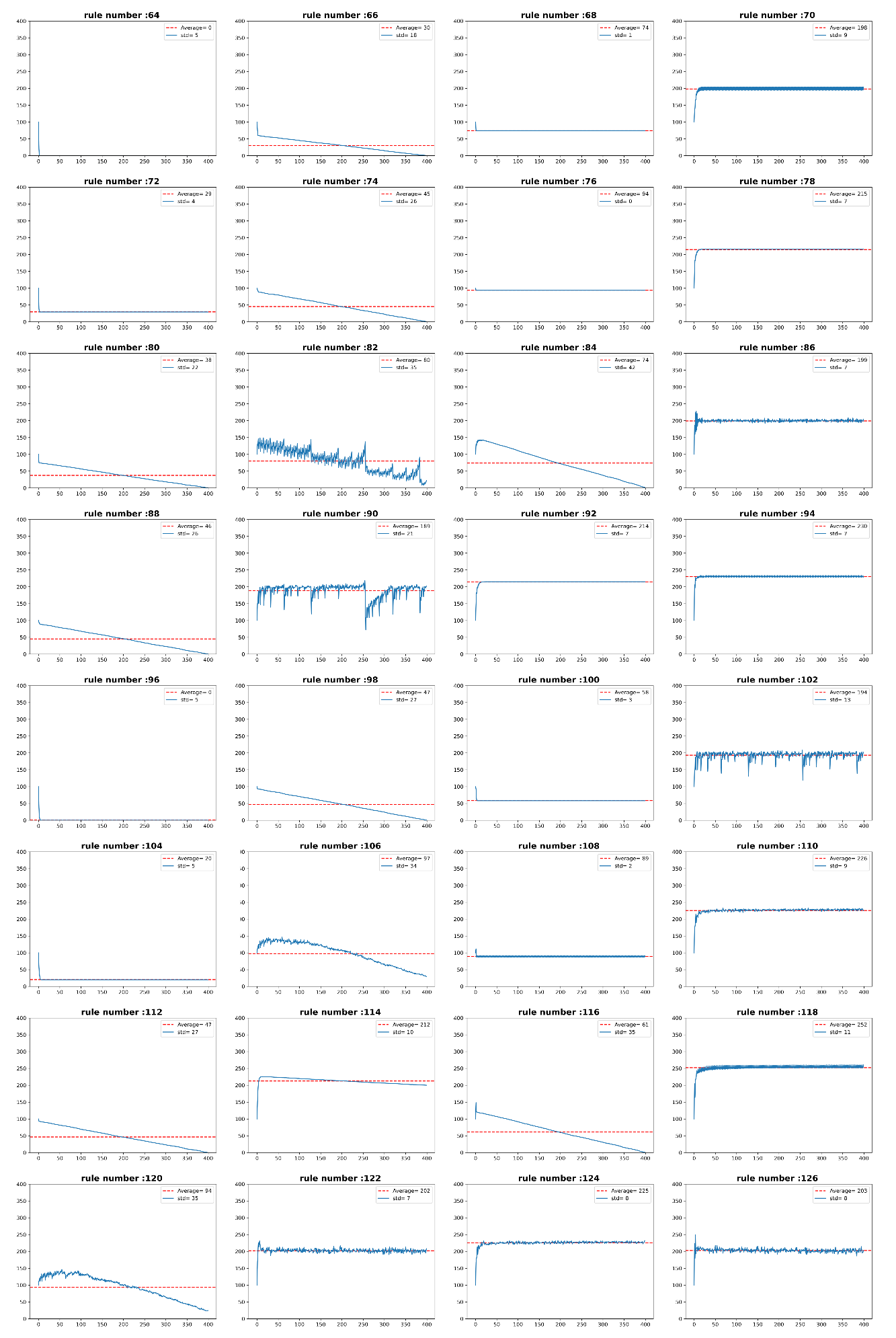


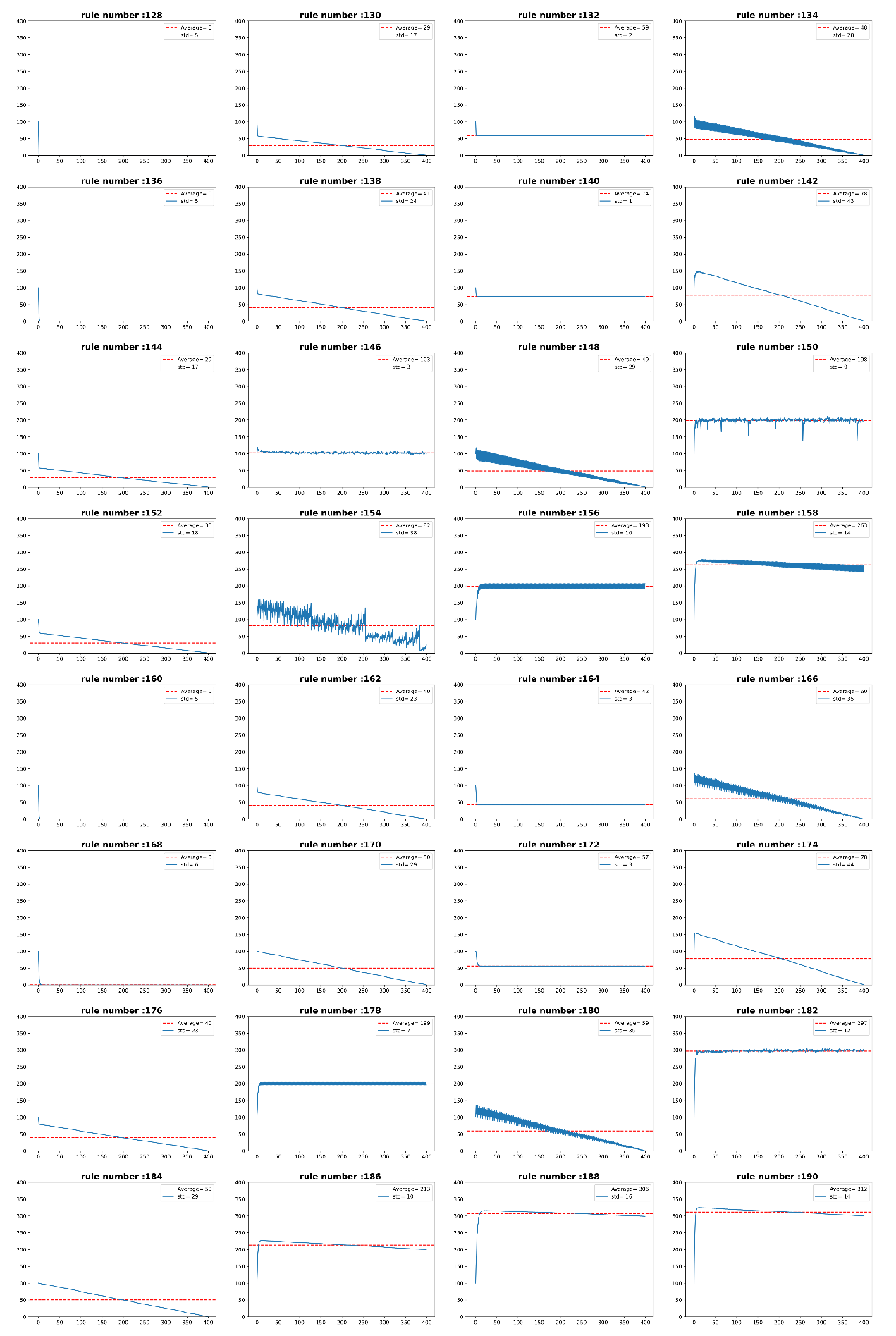


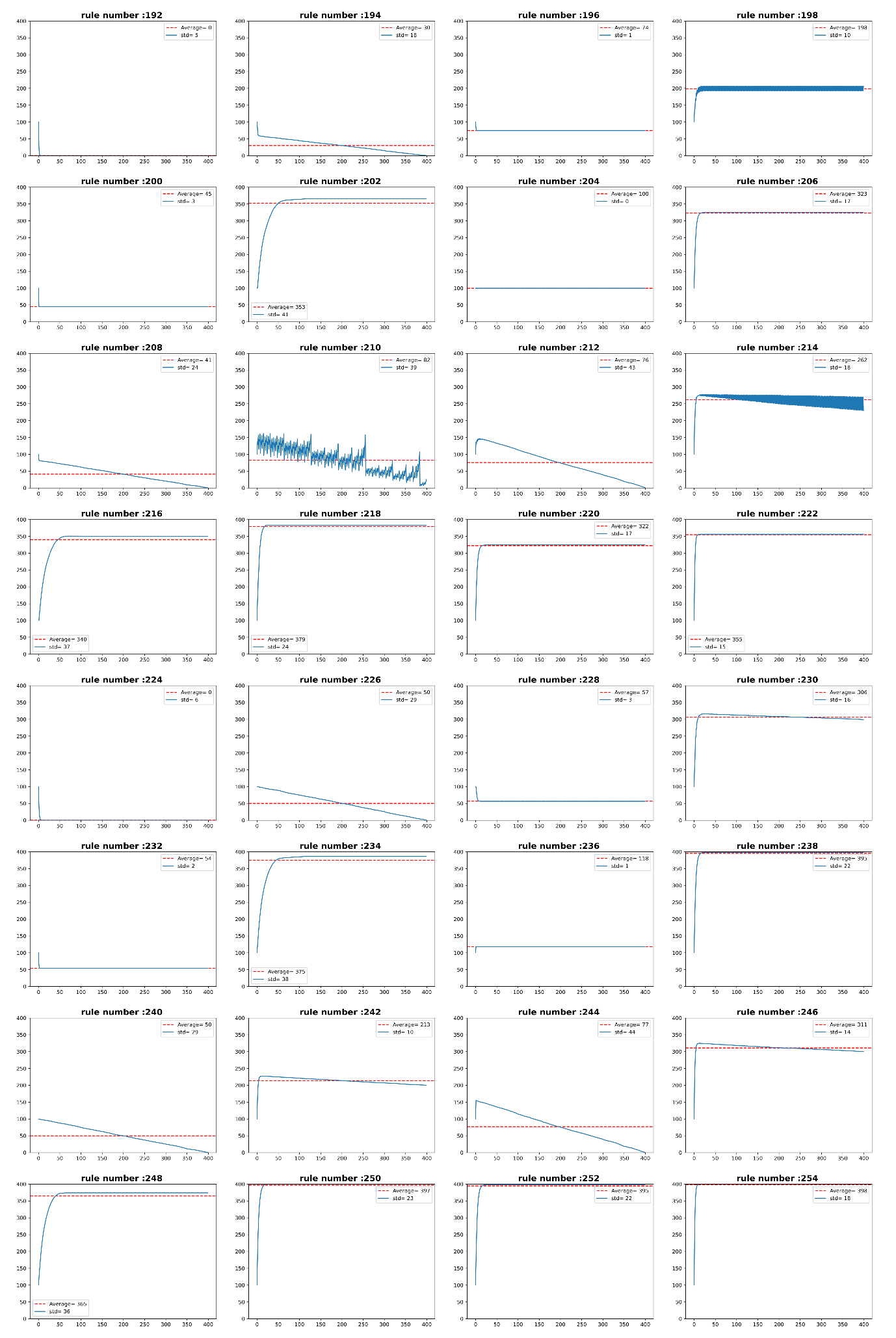


**Fig. S2** **Variation patterns of even ECA rules.** *Variations in the expected number of presence locations over 400 generations according to the ECA even number rules****.*** *A value of 1 is randomly assigned to 100 cells out of 400 cells, and the number of cells having a value of 1 is counted up to 400 generations according to ECA rules. This process is repeated 10 times and the average value is obtained. Cells with a value of 1 correspond to the presence location. The mean value is shown as a red dotted line, and according to the rules, the number of estimated presence locations either maintains the mean value line, decreases, or shows various types of oscillations around the line.*
